# Self-guided quantum state tomography for limited resources

**DOI:** 10.1038/s41598-022-09143-7

**Published:** 2022-03-24

**Authors:** Syed Tihaam Ahmad, Ahmad Farooq, Hyundong Shin

**Affiliations:** Department of Electronics and Information Convergence Engineering, Kyung Hee University, 1732, Deogyeong-daero, Yongin-si, Gyeonggi-do 17104 South Korea

**Keywords:** Quantum information, Qubits

## Abstract

Quantum state tomography is a process for estimating an unknown quantum state; which is innately probabilistic. The exponential growth of unknown parameters to be estimated is a fundamental difficulty in realizing quantum state tomography for higher dimensions. Iterative optimization algorithms like self-guided quantum tomography have been effective in robust and accurate ascertaining a quantum state even with exponential growth in Hilbert space. We propose a faster convergent simultaneous perturbation stochastic approximation algorithm which is more practical in a resource-deprived situation for determining the underlying quantum states by incorporating the Barzilai–Borwein two-point step size gradient method with minimal loss of accuracy.

## Introduction

Quantum state tomography is a field in quantum information that focuses on determining the unknown quantum state. A quantum state can be represented in a Hilbert space, and different known sets of bases are used as projections to extract the information about this quantum state. An enormous ensemble of the unknown state is used to extract the quantum state using the set of known bases. These bases could either be fixed or adaptive depending on the feasibility of the method according to the available resources and required metrics^1–3^. A renowned conundrum in quantum state tomography is the exponential growth of associated parameters with an increase in the dimensionality of the quantum state^4^. This curse of higher dimensionality requires large ensembles of the same state which is practically infeasible. For a *d* dimensional quantum system, $d^{2} -1$ parameters are required to be estimated using the standard quantum state tomography. With an increase in dimension *d*, the required parameters scale as $O\left( d^{2}\right)$. Additionally, more copies of the quantum state are required to achieve better infidelity^5^. The prime goal of the field of quantum state tomography revolves around the improvement of fidelity, for which the field has evolved from standard quantum state tomography methods to better and different approaches ranging from maximum-likelihood estimation, reduced density matrix estimation, variational quantum circuit for state tomography, to recently popular optimization, machine-learning and neural-network approaches for noisy intermediate-scale quantum computing^6–10^.

With the advent of the noisy intermediate-scale quantum computing era, quantum state tomography demands resource-efficient robust methods^11^. One such method was proposed a few years ago as self-guided quantum tomography (SGQT)^8^. One of the prime contributions of SGQT is its robustness which has become a vital part of quantum state tomography since the beginning of noisy intermediate-scale quantum computing era. The robustness of SGQT is a result of its adaptive bases on each iterative step which cannot be attained using fixed basis standard quantum state tomography method i.e., Pauli or computational basis. SGQT uses simultaneous perturbation stochastic approximation (SPSA) which is a pseudo-gradient descent stochastic optimization algorithm rather than an estimation one^12^. It is an iterative method that optimizes the unknown quantum state to the true state by iteratively maximizing the overlap between them^13,14^. This simple iterative algorithm uses only two measurements per iteration regardless of the dimension of the quantum system. Though it has several advantages over other already available methods but the robustness of SGQT to various noise sources is preeminent. However, for higher dimensions, SGQT converges very late to the desired accuracy. Hence, the total number of copies required for tomography increases. Most of the practical applications in quantum information science are performance-demanding and resource-constrained. For example, in practical quantum state tomography the goal is to achieve the best infidelity with a minimum number of quantum state copies in an ensemble.

In this paper, we introduce a new non-monotonic step size method in SPSA to make it resource-efficient. The Barzilai–Borwein (BB) gradient method is efficient for solving large-scale unconstrained problems to modest accuracy and has a great advantage of being easily extended to solve a wide class of constrained optimization problems^15–18^. Our method, a BB-based with clever step-size choice is adapted for SPSA optimization. BB methods are well-known gradient descent based algorithms featuring faster convergence due to clever choice of step length^16,19,20^. Our method successfully overcomes slow convergence in SGQT by explicit use of first-order information of the cost function and the simultaneous implicit incorporation of approximation of Hessian i.e. second-order information of the cost function which is contained in the step size calculation. BB methods have proven to give global convergence for strictly convex quadratic problems and even for non-quadratic cases, the incorporation of BB in a globalization strategy establishes global convergence^15,19,21^. As BB methods yield non-monotonic cost function evaluations, they are combined with line search algorithms i.e. SPSA in our case, along with the maximum of cost functions for previous iterations rather than a monotonic sufficient decreasing conditions for the cost function. This non-monotonic method still gives practically good performance for SGQT with faster convergence in resource deprived tomographic conditions.

Motivated by numerical aspects and practical performance of BB methods, we consider a more practical and resource-efficient environment i.e., self-guided quantum tomography with BB method. In this paper, we extend the BB method for SPSA based self-guided tomography and propose a new step size to accelerate the SPSA method by incorporating accelerated BB step size. Furthermore, by gradient averaging we smooth our convergence graph. Extensive numerical simulations show that our strategies of properly inserting monotone SPSA steps into the non-monotone BB method could significantly improve its performance and the new resulted methods can outperform the most successful iterative optimization algorithm SPSA demonstrated in the recent literature of self-guided tomography for fewer iterations. BB method is a non-monotonic method that gives faster convergence than the normal step size of SPSA. We add monotonic step size condition in traditional BB non-monotonic step size to achieve faster convergence and SPSA like accuracy.

## Method

In this section, we first describe how a standard SPSA algorithm works, then we propose a method to make it more resource-efficient. By utilizing the normal step size with the Barzilai–Borwein step along with gradient averaging and smoothing can achieve better results in terms of resource efficiency. Any arbitrary *d*-dimensional pure quantum state is given by the linear combination

1 $$\begin{aligned} \left| \psi \right\rangle =\sum _{i=0}^{d-1}c_{i}\left| i \right\rangle , \end{aligned}$$

where $\sum _{i}|c_i|^2=1$. To retrieve the information from the quantum system, we need to perform the measurements. The outcomes of the quantum state are obtained by elementary projectors through Born’s rule

2 $$\begin{aligned} f(\phi _{i})=|\left\langle \psi |\phi _{i} \right| \left. |\right\rangle ^{2}, \end{aligned}$$

where $$\sum _{i}\left| \phi _{i} \right\rangle \left\langle \phi _{i} \right| =I$$.

SGQT is a stochastic optimization iterative technique with two measurement settings. At each iteration of SGQT, it performs measurements in $\{f(\phi _{\pm }),I-f(\phi _{\pm })\}$. These measurement settings are used to calculate gradient $g_{k}\left( \phi _{k}\right)$ and the estimated state is updated at each iteration. Starting from a random state $\left| \phi _{0} \right\rangle$, SGQT performs the measurements on $\left| \phi _{k}^{\pm } \right\rangle = \left| \phi _{k}\pm \beta _{k}\Delta _{k} \right\rangle$ at each iteration, where $\Delta _{k}$ is a vector whose entries are chosen uniformly from the discrete sample space $\{-1,+1\}$, and $\beta _{k}= b / \left( k+1\right) ^{t}$ where *k* is the iteration number and (*b*, *t*) are the hyper-parameters. The gradient $g_{k}(\phi _{k})$ of the given direction is then calculated as

3 $$\begin{aligned} g_{k}(\phi _{k})=\frac{f\left( \phi _{k}+\beta _{k} \Delta _{k}\right) -f\left( \phi _{k}-\beta _{k} \Delta _{k}\right) }{2 \beta _{k}}\Delta _{k}. \end{aligned}$$

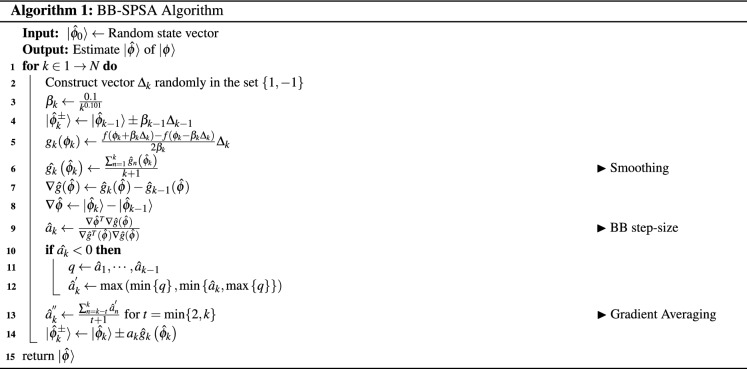


The next estimated state is obtained by the following update rule

4 $$\begin{aligned} \left| \phi _{k+1} \right\rangle = \left| \phi _{k} + \alpha _{k} g_{k} \right\rangle . \end{aligned}$$

Here, gain parameter $\alpha _{k}$ which controls the convergence and decreases with the number of iterations *k* is given by:

5 $$\begin{aligned} \alpha _{k}=\frac{a}{\left( k+1+A\right) ^{s}}. \end{aligned}$$

Here again $\left( a,A,s\right)$ are hyper-parameters for which the optimal parameters are given in^22,23^. At each step, the self-guided tomography is steered by the gradient $g_{k}\left( \phi _{k}\right)$ and will thus converge to the underlying state after a sufficient number of iterations. SGQT claims $s=0.602$, $A=1000$, $a=0.3$, $b=0.1$, and $t=0.101$ as good convergence parameters which worked for SPSA based quantum tomography. However, the asymptotically optimal values suggested by Spall for SPSA, $s=1$ and $t= 1/6$ perform equally well^22^.

The core of the non-monotone methods is based on remembering the data calculated by the preceding iterations. The non-monotone method i.e Barzilai–Borwein method approximates the Hessian matrix and considers the second-order information in the step-size calculation. BB method has already proven to outperform the standard steepest descent methods by Barzilai and Borwein^16^. For an unconstrained optimization problem for minimizing an objective function $f\left( x\right)$ is given by

6 $$\begin{aligned} \min _{x \in {\mathbb {R}} } f\left( x\right) . \end{aligned}$$

The standard steepest descent method calculates the next point using a negative gradient. The next point is given by the update rule

7 $$\begin{aligned} x_{k+1} = x_{k} - \alpha _{k}g_{k}. \end{aligned}$$

For Barzilai–Borwein method, the $\alpha _{k}$ is calculated by solving

8 $$\begin{aligned} \min _{\alpha } \Vert \nabla {x}- \alpha _{k} \nabla {g} \Vert , \end{aligned}$$

where $\Vert \cdot \Vert$ is norm-2 and $\nabla {x} = {x_{k}}- {x_{k-1}}$. By solving this the new BB step size $\alpha _{k}$ is given by

9 $$\begin{aligned} \alpha _{k} = \frac{ \nabla {x} \nabla {g}}{ \nabla {g} \nabla {g}}. \end{aligned}$$

In this paper, we extend a non-monotonic BB method already proven efficient for the steepest descent method for the SPSA algorithm when considering quantum state tomography for fast convergence. We compute the step size using the non-monotonic method suggested by Barzilai and Borwein^16^. To further enhance the results, and make BB method more stable and suitable for our certain problem type, we add gain smoothing and gradient averaging. In the following subsections, we describe the significance of these additions in our proposed method.

Since SPSA relies on a monotone step size $\alpha _{k}$, it has a slow convergence rate, which is not preferred when considering resource efficiency. Moreover, this step size is obtained by experimentation for each problem type. The optimal parameters for quantum state tomography are given in SGQT. The step size parameters for SGQT were obtained by the hit and trial method. However, BB step size does not need hit and trial hyper-parameters. It extracts the second-order information for its following step sizes and hence is adaptive to its convergence regardless of the dimension of the system. To reduce the convergence time, we propose the non-monotone BB method step size as

10 $$\begin{aligned} {\hat{\alpha }}_{k}=\frac{\nabla {\hat{\phi }}^{T} \nabla {\hat{g}}\left( {\hat{\phi }}\right) }{\nabla {\hat{g}}^{T}\left( {\hat{\phi }}\right) \nabla {\hat{g}}\left( {\hat{\phi }}\right) }. \end{aligned}$$

Here $\nabla {\hat{g}}\left( {\hat{\phi }}\right) = {\hat{g}}_{k}\left( {\hat{\phi }}\right) - {\hat{g}}_{k-1}\left( {\hat{\phi }}\right)$ and $\nabla {\hat{\phi }} = {\hat{\phi }}_{k}- {\hat{\phi }}_{k-1}$. We use ${\hat{\alpha }}_{k}$ to indicate it is an estimate rather than a closed form given in SGQT. Sometimes the gain can be negative such that $\nabla {\hat{\phi }}^{T} \nabla {\hat{g}}\left( {\hat{\phi }}\right) <0$ . This is possible because the Hessian of gradient function might include negative eigenvalues at $\nabla {\hat{\phi }}$^24^. Consequently, it is necessary to set closed boundaries around the gain to ensure it is monotonic. Therefore, the current gain becomes

11 $$\begin{aligned} {\hat{\alpha }}_{k}^{\prime }=\max \left\{ \alpha _{\min }, \min \left\{ {\hat{\alpha }}_{k}, \alpha _{\max }\right\} \right\} . \end{aligned}$$

Due to SPSA’s stochastic nature and noisy measurements, the gradients ${\hat{g}}_{k}\left( {\hat{\phi }}_{k}\right)$ can distort the convergence direction. To mitigate such side the effect, the current and the previous *m* gradients are averaged as a gradient estimate at the current iteration

12 $$\begin{aligned} {\hat{g}}_{k}\left( {\hat{\phi }}_{k}\right) =\frac{1}{m+1}\sum _{n=k-m}^{k} {\hat{g}}_{n}\left( {\hat{\phi }}_{k}\right) . \end{aligned}$$

We also add gain smoothing in our method to avoid instability. It essentially averages the gains at the current and last two iterations. So our current gain ${\hat{\alpha }}_{k}^{\prime }$ is updated by gain smoothing as

13 $$\begin{aligned} {\hat{\alpha }}_{k}^{\prime \prime }=\frac{1}{t+1}\sum _{n=k-t}^{k} {\hat{\alpha }}_{n}^{\prime }. \end{aligned}$$

where $$t=2$$.

## Results

In this section, we simulate our results numerically and show the improvement over the SGQT algorithm. We use infidelity as a figure of merit to compare the performance of our algorithm. The infidelity $1-F$ between two pure quantum states is given by

14 $$\begin{aligned} 1-F = 1-|\langle \psi \mid \phi \rangle |^{2}, \end{aligned}$$

where *F* is the fidelity between two quantum states.

**Figure 1:**
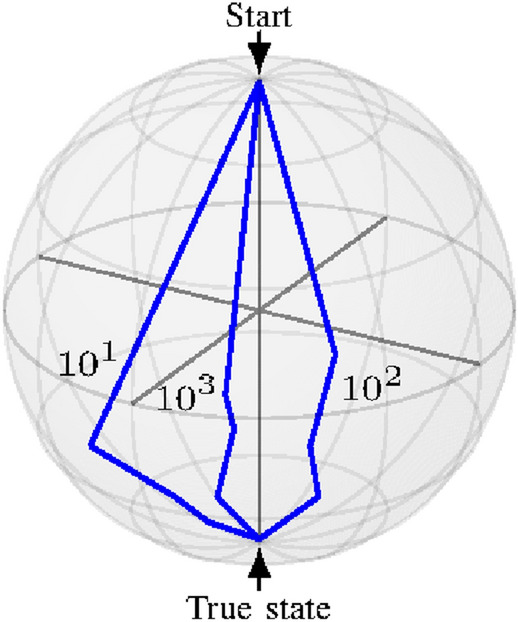
Convergence plot on Bloch sphere for $N=10,100,1000$ for a simple qubit system. We can see that BB-SPSA converges faster with increase in number of measurements per setting.

First, we performed our numerical analysis for a single qubit case and experiment with the number of copies to observe the difference of performance with the number of copies. Through experimentation, we found that by increasing the number of copies per iteration, the algorithm converges faster as shown in Fig. 1.

**Figure 2:**
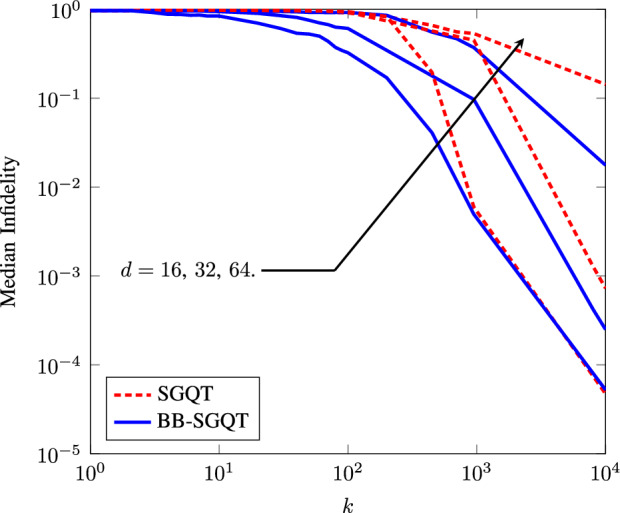
Average infidelity plots for qubits $d=16{,}32{,}64$ obtained from 100 randomly generated qubits using Haar measurement. Blue lines show the median infidelity plots for our algorithm, and red dotted lines show the same plots for SGQT. Here, we choose $N=10^4$ number of measurements per iteration.

We observe that our algorithm performs almost equally well as SGQT with a marginal difference for $d=2$ but our algorithm is more advantageous for higher dimensions. We performed our experiments by taking median infidelity of our algorithm and SGQT for 100 randomly generated quantum states using Haar measure for each dimension $d=16,32,64$. In our simulations, we refer *N* as the number of measurements per estimate; *M* as the number of estimates per iteration; and *k* as the number of iterations. Hence, the total number of measurements performed after *k* iterations is $N_{\mathrm {tot}}=kMN$. It is to be noted as one of the advantages of SGQT over standard finite gradient estimation where $M=2d$ for *n* pure qubits, *d* is the real dimension of the state space given as $d=2(2^n -1)$, which grows exponentially^8^. For SGQT, $M=2$ which is fixed for any dimensional quantum system. Thus, we restrict our attention to *N* and *k*, with the understanding that $N_{\mathrm {tot}}=2Nk$. We choose $N = 10^4$ to have a fair comparison with self-guided tomography. From experimentation, we observe our proposed method outperforming SGQT for resource-constrained quantum estimation. Even for lower dimensions, our algorithm almost works equally well with marginal difference in performance. We observe our algorithm converging noticeably faster for higher dimensions as compared to convergence observed in SGQT given in Fig. 2. The scaling of SPSA algorithm used in SGQT was analyzed by Spall^22,23^. This convergence analysis implies that the infidelity decreases at the rate $O\left( \frac{d^{\gamma }}{N_{\mathrm {tot}}}\right)$, where *d* is the dimension of the quantum system and $\gamma$ is problem-dependent^8^. In SGQT, the asymptotic scaling of infidelity is found to be $O\left( \frac{d^{\gamma }}{N_{\mathrm {tot}}}\right)$ with $\gamma \in \left( 1.02,1.35\right)$. We present the infidelity improvement scaling of BB-SGQT over SGQT in Table 1. BB method finds the solution faster than the SPSA with a small sacrifice in accuracy.

**Figure 3:**
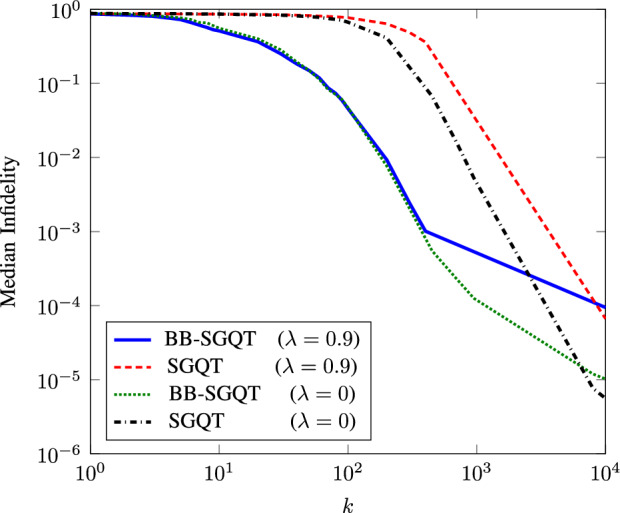
Average infidelity plots for $d=8$ with $90\%$ depolarizing noise (red and blue) and without noise (green and black) are plotted by taking the average for 100 randomly generated qubits using Haar measurements. Red and black represent standard SGQT graphs, while blue and green color is for our BB-SGQT method. Here, we fixed $N=10^4$ the number of measurements per iterations for each method.

To demonstrate that our efficient method is equally robust, we use doubly stochastic matrix $\Lambda$. This doubly stochastic matrix models the depolarizing noise in a faulty measurement device^25^. The matrix $\Lambda$ is defined as

15 $$\begin{aligned} \Lambda _{i,j}={\left\{ \begin{array}{ll} 1-\lambda +\lambda /d, &{}\quad \text {when}\;\;\; i=j \\ \lambda /d, &{}\quad \text {otherwise}, \end{array}\right. } \end{aligned}$$

where $\lambda$ corresponds to the strength of noise in measurement outcomes. We perform our experiments for $d=8$ with and without depolarizing noise i.e., $\lambda =0.9$. The settings are similar as used in aforementioned case. We can clearly see that our algorithm does not loses its robustness by introducing high noise model as shown in Fig. 3.

Conjugate gradient-descent (CGD) and projected gradient-descent (PGD) based approaches have been also used for quantum state tomography^26,27^. These approaches are used as post-processing techniques on maximum likelihood estimation or standard quantum tomography to give better fidelity. However, these approaches cannot be extended to be used within the experiment but just as post-processing techniques. These methods require a well-defined loss function. Due to the unknown target state to be measured, a well-defined loss function cannot be attained in quantum state tomography and hence gradient cannot be calculated. Hence, we cannot apply gradient-descent algorithm solely in quantum state tomography. Nevertheless, SPSA algorithm, a pseudo-gradient descent technique can be applied, which approximates the gradient from noisy loss function measurements and performs very well in quantum state tomography.

**Figure 4:**
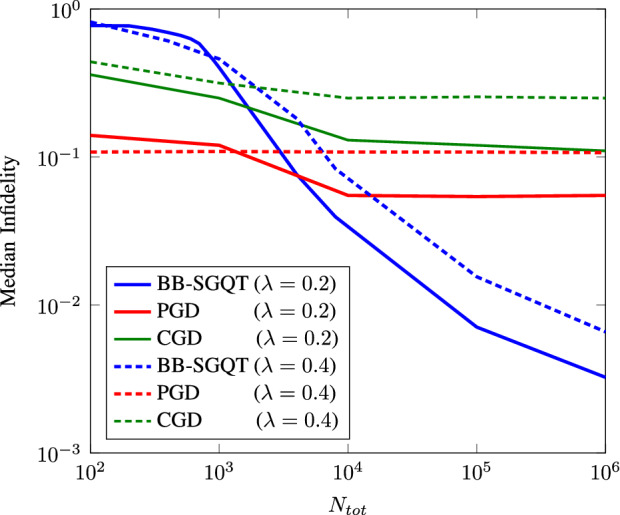
Average infidelity plots for $d=4$ with $20\%$ and $40\%$ depolarizing noise (solid and dashed) are plotted against total resources $N_{tot}$ by taking the average for 100 randomly generated qubits using Haar measurements. Blue, red and green represent BB-SGQT, PGD and CGD respectively.

We also compare our method with other already suggested gradient-descent based methods such as conjugate gradient-descent and projected gradient-descent with maximum likelihood estimation to emphasize the robustness advantage over other optimization techniques. We run the experiments for $d=4$ with $\lambda =0.2$ (low) and $\lambda =0.4$ (high) depolarizing noise to see the practical quantum state estimation results over total number of resources used in our simulation as shown in Fig. 4. We observe that BB-SGQT keeps on improving the fidelity of a quantum state with increasing number of resources, even in high depolarizing noise experiment. However, other methods are saturated and cannot give any better fidelity even with high number of resources and low noise, making them impractical for experimental quantum tomography.

**Table 1:** Scaling of performance in BB-SGQT vs SGQT with qubit dimension.

	*k*	BB-SGQT	SGQT	**Improvement**
$d=16$	$10^1$	$8.39 \times 10^{-1}$	$9.75 \times 10^{-1}$	$\times 1.16$
	$10^2$	$3.24 \times 10^{-1}$	$9.39 \times 10^{-1}$	${\times 2.89}$
	$10^3$	$4.93 \times 10^{-3}$	$5.94 \times 10^{-3}$	${\times 1.20}$
	$10^4$	$5.23 \times 10^{-5}$	$4.73 \times 10^{-5}$	Marginal difference
$d=32$	$10^1$	$9.41 \times 10^{-1}$	$9.74 \times 10^{-1}$	${\times 1.03}$
	$10^2$	$6.07 \times 10^{-1}$	$9.25 \times 10^{-1}$	$\times 1.52$
	$10^3$	$9.66 \times 10^{-2}$	$4.48 \times 10^{-1}$	${\times 4.63}$
	$10^4$	$2.50 \times 10^{-4}$	$7.29 \times 10^{-4}$	$\times 2.91$
$d=64$	$10^1$	$9.54 \times 10^{-1}$	$9.55 \times 10^{-1}$	Same performance
	$10^2$	$9.18 \times 10^{-1}$	$9.20 \times 10^{-1}$	Same performance
	$10^3$	$3.70 \times 10^{-1}$	$5.33 \times 10^{-1}$	$\times 1.44$
	$10^4$	$1.76 \times 10^{-2}$	$1.42 \times 10^{-1}$	${\times 8.06}$

## Discussion

With the promising practicality of noisy intermediate-scale quantum computers, robust and resource-efficient quantum state tomography for higher-dimensional quantum systems has become inevitable^9^. All the recent state tomography methods have failed to provide either robustness or resource efficiency. Likewise, self-guided tomography provides robustness but is not suitable for resource-constrained quantum state tomography. However, the proposed Barzilai–Borwein method has proven to overcome this deficiency of self-guided tomography, particularly for higher-dimensional quantum states. Also, hyper-parameters required by step size used in SGQT were obtained using the hit and trial method as compared to our method that adaptively computes its step size for any dimensional quantum system without the need for hyper-parameters.

Even though our proposed method is practically more feasible than SGQT when there are fewer copies, it still does not outperform SGQT in the long run when the resources are enough. After performing our empirical experiments, we noticed that our method, though resource-efficient, loses its accuracy as compared to self-guided tomography in the long run. It performs equally well for higher-dimensional quantum systems with marginal accuracy difference to standard self-guided tomography.

Self-guided tomography has already been experimentally implemented^11,28^. Standard SGQT relies on using entangled basis on each iteration which is very hard to implement practically. However, it has been shown that by approximating the entangled basis from the combination of Pauli basis which are easy to prepare, SGQT still outperforms the standard quantum state tomography algorithm in a noisy environment^28^. We know that Pauli measurement-based SGQT gives a poor performance as compared to standard SGQT yet it is advantageous over standard quantum state tomography methods in terms of fidelity, post-processing cost and robustness^28^. With a small change in step size in self-guided tomography, our method can also be achieved in experiments. Hence, making it totally resource-efficient, robust, and most importantly, practical.

## Data Availability

The data generated from empirical results and the source code that support the findings of this study are available from the corresponding author upon reasonable request.
